# Effects of subjective and objective autoregulation methods for intensity and volume on enhancing maximal strength during resistance-training interventions: a systematic review

**DOI:** 10.7717/peerj.10663

**Published:** 2021-01-12

**Authors:** Stian Larsen, Eirik Kristiansen, Roland van den Tillaar

**Affiliations:** Department of Sport Sciences and Physical Education, Nord University, Levanger, Norway

**Keywords:** Autoregulation, Maximal strength, Resistance training

## Abstract

**Background:**

Maximal strength is a critical determinant of performance in numerous sports. Autoregulation is a resistance training prescription approach to adjust training variables based on the individuals’ daily fluctuations in performance, which are a result of training-induced fitness and fatigue, together with readiness from daily non-training stressors.

**Objective:**

This review aimed to summarise the effects of different subjective and objective autoregulation methods for intensity and volume on enhancing maximal strength.

**Materials and Methods:**

A comprehensive literature search was conducted through SPORTDiscus, PubMed and Google Scholar. Studies had to meet the following criteria to be included in the review: (1) estimation of 1-RM or a 1-RM test for both pre-test and post-test to measure progression in strength assessment during the training intervention, (2) a training comparison group, (3) participants were healthy, (4) the article had a detailed description of training intensity, training volume, and training frequency during the training intervention, (5) the training intervention lasted for more than four weeks, (6) studies with objective autoregulation methods utilised a validated measuring tool to monitor velocity, (7) English-language studies.

**Results:**

Fourteen studies met the inclusion criteria, comprising 30 training groups and 356 participants. Effect size and percentage differences were calculated for 13 out of 14 studies to compare the effects of different training interventions. All autoregulation training protocols resulted in an increase in 1-RM, from small ES to large ES.

**Conclusion:**

Overall, our findings suggest that using both subjective autoregulation methods for intensity, such as repetitions in reserve rating of perceived exertion and flexible daily undulation periodisation, together with objective autoregulation methods for autoregulation intensity and volume, such as velocity targets and velocity loss, could be effective methods for enhancing maximal strength. It is speculated that this is because the implementation of autoregulation into a periodised plan may take into account the athletes’ daily fluctuations, such as fluctuations in fitness, fatigue, and readiness to train. When training with a validated measuring tool to monitor velocity, this may provide objective augmented intra- and interset feedback during the resistance exercise who could be beneficial for increasing maximal strength. Coaches, practitioners, and athletes are encouraged to implement such autoregulation methods into a periodised plan when the goal is to enhance maximal strength.

## Introduction

Resistance training is a training method used to strengthen and increase the size of the skeletal muscles to achieve better performance in everyday activities and sports. Some documented effects of resistance training are changes in muscular hypertrophy, strength, and power (DeLorme, 1945; Folland & Williams, 2007). It is well documented that the manipulation of the training variables of volume, intensity, and frequency during resistance training can affect the physiological and bodily adaptations such as muscle strength and hypertrophy (Grgic et al., 2018; Ralston et al., 2017; Schoenfeld, Grgic & Krieger, 2019; Schoenfeld, Ogborn & Krieger, 2016).

Determining the intensity for a set is often based on direct one repetition maximum (1-RM) assessment. This is done with the athlete or participant exercising on a percent-based load from a previous lifted 1-RM as a reference (Jovanović & Flanagan, 2014). However, when using this method, 1-RM may not be relative to the current maximal strength level of individuals. Helms (2017) reviewed physiological variables and hormonal and muscle damage biomarkers that correlated with resistance performance. These variables were salivary testosterone levels, salivary cortisol levels, epinephrine, norepinephrine, heart rate variance and creatine kinase. Therefore, it may be that the fluctuations of physiological variables such as hormonal and muscle damage biomarkers could affect daily or weekly strength fluctuations. These strength fluctuations are often caused by training, which could increase fatigue or fitness, such as maximal strength due to continuous resistance training (Eston & Evans, 2009). In its most basic form, this is known as the fitness-fatigue model, which was originally developed by Banister et al. (1975). However, Banister et al. (1975) model just account for that changes in performance could be attributed only to training (Greig et al., 2020). This is not the case in the real world, because daily stressors such as nutrition, sleep, illness could influence performance. Greig et al. (2020) argued that this can be viewed synonymously with the concept of readiness. Therefore, the three key concepts of daily fluctuation in performance could be the sum of fitness, fatigue, and readiness. Where fitness could be described as positive effects on performance and adaptations from training. Fatigue could be described as the negative effects on performance from training, and readiness is the stochastic variation in performance attributed to non-training daily stressors (Greig et al., 2020).

Based on that fluctuations in performance could occur, different training prescription methods, referred to as autoregulation has become popular to increase maximal strength (Helms et al., 2017a). Autoregulation is a resistance training prescription approach to adjust the training variables intensity, volume and frequency based on the daily individual fluctuations in fitness, fatigue and readiness of the athlete. To enhance strength performance, it could be worth to provide an overview regarding the effects of different autoregulation methods on enhancing maximal strength during resistance-training interventions.

### Subjective autoregulation methods

Borg (1970) introduced the first rating of perceived exertion (RPE) scale, which rated intensity based on the subjective perception of intra-set effort. The Borg RPE scale was originally designed for endurance sports and ranged from 6 to 20 to reflect the heart rate (Borg, 1970). In Borg (1982), modified the scale to apply from 1 to 10 (Borg CR10 RPE scale), where 1 was ‘nothing at all’ and 10 was ‘extremely strong, almost max. However, studies found that the participants failed to lift the weights when they rated the intensity to be 6.8–8.1 RPE (Pritchett et al., 2009; Shimano et al., 2006). Therefore, the Borg CR10 RPE scale did not appear to be reliable in resistance training. Halperin & Emanuel (2020) addressed that there are several definitions (Abbiss et al., 2015; Marcora, 2009; Pageaux, 2016) and measurement instruments of perceived effort (Abbiss et al., 2015; Marcora, 2009; Venhorst, Micklewright & Noakes, 2018), which may lead to confusion and hinder measurement validity. However, in Tuchscherer (2008), modified the Borg CR10 RPE scale, whereby RPE was determined by how many repetitions in reserve (RIR) the participant felt he or she had left before reaching failure. The problem with rating RPE wrong was identified by Hackett et al. (2012), who compared the Borg CR10 RPE scale with an RIR scale using bodybuilders. They found that there was a 0.63 limit of agreement between rating the Borg CR10 and RIR when squats and bench press were performed, the authors also found that accuracy improved closer to failure. Therefore, Zourdos et al. (2016b) conducted research on RIR-based RPE. Where they took the concept that Hackett et al. (2012) had shown and combined it with the RPE scale of Tuchscherer (2008), and thereby investigated the correlation between the RIR-based RPE scale and average velocity. The study was carried out with both experienced and novice lifters. The mean RPE rating for experienced and novice lifters was 9.8 and 8.96, 7.87 and 7.46, 5.18 and 4.89, and 3.54 and 3.73 for 1-RM, respectively, and 90%, 75% and 60% of 1-RM. For experienced and novice lifters, there was a strong inverse relationship between average velocity and RPE across all percentages (*R* = −0.88 and *R* = −0.77). Furthermore, Helms et al. (2017b) compared both average concentric velocity and RIR based RPE in the squat, bench press, and deadlift for powerlifters. They found strong relationships between percentage 1-RM and RPE for each lift (*r* = 0.88–0.92). It was concluded that RPE was a reliable tool for prescribing intensity for the squat, bench press and deadlift for powerlifters. However, RIR has been reported to be less accurate among untrained subjects (Steele et al., 2017), and in sets including a high amount of repetitions (Hackett et al., 2017; Zourdos et al., 2019). Therefore, it is being speculated that RIR based RPE could be better for prescribing training intensity among experienced resistance-trained participants during sets with a relatively low number of repetitions.

Training volume is another training variable that has been shown to impact maximal strength adaptations due to continuous resistance training (Robbins, Marshall & McEwen, 2012). Gonzalez-Badillo et al. (2005) investigated the effect of three resistance training volumes on maximal strength in the snatch, clean and jerk and squat, during a 10-week training intervention on 51 trained junior lifters. The investigators found that moderate volume (2,481 repetitions) produced a superior increase in strength compared to the training group that trained with low volume (1,923 repetitions) and high volume (3,030 repetitions). Therefore, autoregulation for volume could be necessary when training with an autoregulation method for intensity. Helms et al. (2018b) investigated how a RIR-based RPE strategy could impact the volume performed by powerlifters. To investigate this, twelve participants performed the back squat, bench press and deadlift three times per week, where the workouts were performed in a hypertrophy, power and strength order. During each workout, participants performed one or two top sets for a prescribed number of repetitions at a target RPE. If the RPE score was lower than the target RPE on the first top set, a second top set was performed with an increased load to reach the target RPE Thereafter, back-off sets were performed at a reduced load (intensity-decrease at 6%, 4% and 2%) for the same number of repetitions. When the participants rated the RPE to be the same or greater at the back-off sets compared with the top set, the workout stopped. The findings were that the weekly combined relative volume for back-off sets performed during the back squat, bench press and deadlift was different between weeks 1, 2 and 3 when different percentage RPE stops were used. Combined back squat and deadlift volume was significantly higher with a 6% rather than a 2% RPE stop. Bench press volume was significantly different for each training week, where 6% was the highest volume, followed by 4% and 2% RPE stop. The conclusion was that RPE stops could be an effective way to autoregulate volume because this could dictate the number of sets performed. However, no studies have investigated the effects of RPE stops on 1-RM compared to a fixed volume programme.

RPE and RIR are not the only subjective autoregulation methods for prescribing intensity and volume. DeLorme (1945) experimented with resistance training for rehabilitating injuries in servicemen. The training protocol consisted of multiple sets of resistance exercises where patients lifted their 10-RM (Todd, Shurley & Todd, 2012). DeLorme (1945) later modified the resistance programme to include three progressive sets of 10 repetitions. The resistance programme was called progressive resistance exercise (Delorme, Schwab & Watkins, 1948). A weakness was that this training method did not allow for individual progression in strength. Therefore, Knight (1979) addressed this problem with a programme that he called daily adjustable progressive resistive exercise. During the first set, the patient performed 10 repetitions against one-half of the estimated load of working sets. During the second set, the patient performed six repetitions against three-quarters of working weights. During the third set, the patient trained at full working weight with maximum repetitions. During the fourth set, the intensity was determined by repetitions performed during the third set. The number of repetitions performed during the fourth set informed the load to use during the following resistance training session. If less than two repetitions were performed in set four, then a decreased load of 2.5–5 kg was used in the following resistance training session. On the contrary, if 3–4 repetitions were performed, the same load was kept for the following workout. Finally, when 5–6, 7–10 or 11+ repetitions were performed, the load was increased by 2.5–5, 5–7 or 5–10 kg in the following resistance training session (Knight, 1979). The daily adjustable progressive resistive exercise training method was later modified by Siff (2000) and outlined for increasing muscular strength. This training method was called autoregulatory progressive resistance exercise. The method for autoregulatory progressive resistance training is to perform the last set in an exercise to failure. The number of repetitions performed dictates the intensity for the next workout (Mann et al., 2010).

Another way to prescribe a resistance plan is by changing the muscular stimulus between sessions. This is called daily undulating periodisation or nonlinear periodisation, and allows an athlete to train hypertrophy, strength, and power in the same week (McNamara & Stearne, 2010). This training method has been shown effective for increasing muscular strength and endurance (Peterson et al., 2008; Rhea et al., 2002; Rhea et al., 2003). Kraemer & Fleck (2007) introduced the continuation of daily undulating periodisation, named flexible nonlinear periodisation. This training method allows the athlete to choose the training stimulus immediately before the workout, based on physiological and mental readiness. This could be done by rating readiness on a 0–10 subjective scale, and thereby choose the training stimulus to be either hypertrophy, strength, or power specific for the training session (McNamara & Stearne, 2013).

### Objective autoregulation methods

The development of modern technology enables objective autoregulation methods for measuring intensity, thereby adjusting both intensity and volume. Velocity-based resistance training (VBT) is a training method that uses validated measuring tools, such as accelerometers, high speed/velocity cameras, linear position transducer, or velocity transducers to track the movement velocity of an exercise (Jovanović & Flanagan, 2014). VBT at its most basic, can accessory traditional percentage-based training, which means for example augmented verbal or visual feedback, to enhance motivation(Weakley et al., 2019b, 2019a). Another way of implementing VBT into a resistance training programme is to support the prescription of intensity, sets, and a number of repetitions (Weakley et al., 2020). To implement a full velocity-based programme, a load-velocity profile could either be individualised or used at a group-level (Moore & Dorrell, 2020). Early research on the load-velocity relationship investigated group equations, which could be implemented into groups and thereby gave researchers, coaches and practitioners average group-based velocity profiles (Moore & Dorrell, 2020; Sanchez-Medina & Gonzalez-Badillo, 2011). While in later years, the load velocity profile has been frequently individualised since the group profile could be accurate for some, but not for others (Moore & Dorrell, 2020).

The literature regarding quantifying 1-RM with the load-velocity relationship has varying and divergent findings. In several studies, the load-velocity relationship has been shown to be a reliable measure for intensity within a training session when using both relative load or daily 1-RM (Bazuelo-Ruiz et al., 2015; Conceicao et al., 2016; Gonzalez-Badillo & Sanchez-Medina, 2010; Jidovtseff et al., 2011; Munoz-Lopez et al., 2017; Picerno et al., 2016). These results suggest that barbell velocity can predict the intensity (i.e., 1-RM or % of 1-RM) (Garcia-Ramos et al., 2017). Therefore, Banyard et al. (2018) investigated peak velocity, mean propulsive velocity, and mean velocity in the development of load-velocity profiles in the back squat between different training sessions. In the study, resistance-trained males performed a 1-RM back squat and then three 1-RM trials with 48 h of rest between further trials. In 1-RM trials, the participants lifted 20%, 40%, 60%, 80%, 90% and 100% of 1-RM. The finding was that peak velocity was highly reliable across all six loads between the training sessions. The authors concluded that peak velocity at 20–100%, mean propulsive velocity at 20–90%, and mean velocity at 20–90% of 1-RM were reliable and could be used to develop a load-velocity profile using linear regression. Also, they concluded that load-velocity profiles could be used to monitor changes in velocity and therefore employed as a method for adjusting sessional training loads according to the daily fluctuations of the athlete. Furthermore, Fahs, Blumkaitis & Rossow (2019) demonstrated that velocity profiles must be exercise-specific when they investigated average concentric velocity in the back squat, bench press, deadlift, and overhead press at different percentages of 1-RM. Another potential benefit of VBT may be that the load-velocity profile has been shown to remain unchanged despite an increase in strength (Balsalobre-Fernández, García-Ramos & Jiménez-Reyes, 2018), and have therefore been theorised to be an objective autoregulation method for prescribing training intensity (Moore & Dorrell, 2020). It has also been demonstrated that the velocity at a given percentage of 1-RM can shift because of fatigue (Hughes et al., 2019; Vernon, Joyce & Banyard, 2020), or after a power-oriented training programme (Pérez-Castilla & García-Ramos, 2020). To avoid such inaccuracies, Weakley et al. (2020) recommended in their review to periodically assess the load-velocity relationship for accurate prescription of relative loads. However, several studies have presented less favourable findings with respect to quantifying 1-RM using the load-velocity relationship (Banyard, Nosaka & Haff, 2017; Hughes et al., 2019; Jukic et al., 2020; Lake et al., 2017; Ruf, Chéry & Taylor, 2018). In these studies, the use of different regression equations to predict 1-RM from the load-velocity relationship mostly tended to overestimate the predicted 1-RM, when compared to the real 1-RM lifted.

According to Weakley et al. (2020), there are two common ways to prescribe training intensity and the intra-set volume for VBT. (1) Velocity targets + velocity loss threshold to terminate the set, (Galiano et al., 2020; Pareja-Blanco et al., 2020; Pareja-Blanco et al., 2017a, 2017b; Rodríguez-Rosell et al., 2020; Sánchez-Moreno et al., 2020). (2) Set average velocity + velocity loss threshold (Dorrell, Smith & Gee, 2019; Orange et al., 2019; Shattock & Tee, 2020). These methods differ, where the set average velocity + velocity loss thresholds start with an external load prescribed from a load-velocity profile, which needs to decrease until a pre-determined velocity zone for the set to end. While for the velocity targets + velocity loss thresholds, the set starts with a prescribed velocity or velocity range, before the percentage of velocity loss determines the set termination (Weakley et al., 2020).

Since studies suggest that both subjective and objective autoregulation methods could be used to prescribe training variables based on the daily fluctuations of the athlete, this study aimed to conduct a systematic review of the literature regarding the effects of subjective and objective autoregulation methods for intensity and volume on enhancing maximal strength during resistance-training interventions.

## Methods

### Data collection

**Figure 1:**
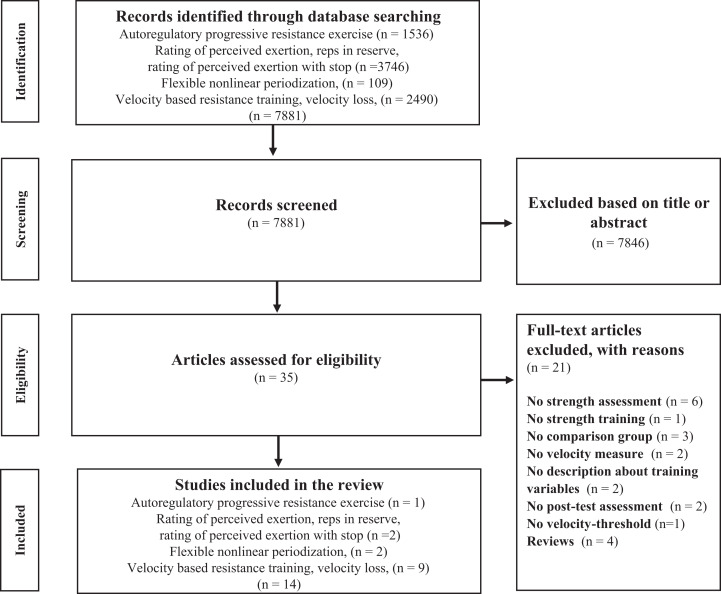
Flow diagram of the search process.

The systematic review was based on original research. A literature search for journals in the English language was conducted up to August 2020. No time window was used as search criteria during the data collection. Data were collected from the following databases: SPORTDiscus, PubMed and Google Scholar (Fig. 1). The following keywords were used to carry out the search: Autoregulation, Rating of perceived exertion, repetition in reserve, RPE-stop, autoregulatory progressive resistance exercise, flexible nonlinear periodisation, velocity-based resistance training, velocity loss, linear position transducer, linear velocity transducer, one repetition maximum. Search terms were modified to fit the requirements of the database used.

For the database PubMed, the following keywords were used: ‘autoregulatory progressive resistance exercise,’ ‘rating of perceived exertion,’ ‘reps in reserve,’ ‘RPE-stop,’ ‘one repetition maximum,’ ‘flexible nonlinear periodisation,’ ‘velocity-based training,’ ‘velocity-based resistance training,’ ‘velocity loss,’ ‘linear position transducer,’ ‘linear velocity transducer,’ with the search mode: ‘advanced search, all fields,’ which resulted in 208 hits.

The original research had to meet the following criteria to be included in the review: (1) estimation of 1-RM or a 1-RM test for both pre-test and post-test to measure progression in strength assessment during the training intervention, (2) a training comparison group, (3) participants were healthy, (4) the article had a detailed description of training intensity, training volume, and training frequency during the training intervention, (5) the training intervention lasted for more than four weeks, (6) studies with objective autoregulation methods utilised a validated measuring tool to monitor velocity, (7) English-language studies. The data search was conducted by the first author: Stian Larsen. After reviewing the literature, 14 studies met the inclusion criteria. For simplicity, studies which use barbell velocity to monitor training prescription are referred to as objective autoregulation methods. All other methods in this review are referred to as subjective autoregulation methods. Pre-test and post-test values from Shattock & Tee (2020) and Helms et al. (2018a) were retrieved from mail correspondence with the corresponding authors.

Studies are presented in Table 1 (subjective autoregulation methods) and Table 2 (objective autoregulation methods), which show the following: (1) References. (2) Training group, sample size within the training group, age expressed as mean ± standard deviation (SD). (3) Duration of the training intervention, weekly training sessions. (4) Resistance-training exercise. (5) Experimental group’s pre- and post-test strength assessment expressed as mean ± SD in kilograms (kg). (6) Comparison group’s pre-and post-test strength assessment expressed as mean ± SD in kg. (7) Both training groups’ progression from pre-test to post-test expressed in percent (%). (8) Both training groups’ progression from pre-test to post-test expressed in effect size (ES). ESs in Tables 1 and 2 were calculated using Cohen’s d, $\textstyle{{m1 - m2} \over {\; SD\; pooled}}$. An ES from 0.2 to 0.5 is considered small. An ES from 0.5 to 0.8 is considered moderate. An ES > 0.8 is considered large (Cohen, 1988). (9) Other variables tested in the pre-test and post-test. (10) Sets × repetitions matched between groups.

**Table 1:** Overview of intervention studies assessing a subjective autoregulation training-method for manipulating training-intensity or training-volume upon 1-RM performance.

Subjective autoregulation methods									
Reference	Training-group; Sample size (n); age (year)	Duration weeks × sessions per week	Resistance training exercise	Experimental pre: post: strength assessment (kg)	Comparison pre: post: strength assessment (kg)	Progression percent from pre: to post: both training groups (%)	Effect size from pre: to post: both training groups (Cohen’s d)	Other variables measured pre: and post:	Sets × reps equal between groups
**Rating of perceived exertion, reps in reserve**									
Helms et al. (2018a, 2017)	RT male RPE: *n* = 10 PB: *n* = 11 age: 22.4 ± 5.5	8 × 3	Back squat Bench press	RPE pre: 143.7 ± 24.9, 120.9 ± 19.3 RPE post: 160.7 ± 28.4, 131.6 ± 19.5	PB pre: 139.2 ± 18.2, 113.9 ± 18.7 PB post: 153.1 + 16.6, 123.5 ± 17	RPE: 11.8, 8.9 PB: 10, 8.4	RPE: 0.64, 0.55 PB: 0.80, 0.52	Post: m. pectoralis; m. vastus lateralis muscle thickness at 50% and 70% of femur length	Yes
Graham & Cleather (2019)	RT male RIR: *n* = 15 PB: *n* = 16 age: 28.1 ± 5.5	12 × 2	Front squat Back squat	RIR pre: 120.7 ± 26.3, 141.2 ± 29.4 RIR post: 134.8 ± 26.1, 156.4 ± 29.8	PB pre: 111.3 ± 19.6, 129.1 ± 21.3 PB post: 120.6 ± 18.3, 138.2 ± 19.5	RIR: 11.7, 10.8 PB: 8.4, 7	RPE: 0.53, 0.51 PB: 0.49, 0.45	Countermovement jump; sprint 10, 20, 40 metres	Yes
Shattock & Tee (2020)	Rugby players RT male. VBRT: *n* = 10 RPE: *n* = 10	Maximal strength: 6 × 4 Strength speed: 6 × 3	Back squat Bench press	VBRT -> RPE pre: 137 ± 22.5, 102.2 ± 20 VBRT -> RPE 6-week post: 149 + 21.1, 112.3 ± 19.6 VBRT -> RPE 12-week post: 153.3 ± 20.7, 115.3 ± 19.5	RPE -> VBRT pre: 152.5 ± 26.6, 116.5 ± 17.3 RPE -> VBRT 6-week post: 158.5 ± 26, 122 ± 16.9 RPE->VBRT 12-week post: 167.3 ± 24.6, 128 ± 15.3	VBRT -> RPE 6-week post: 8.8, 9.9 VBRT-> RPE 12-week post: 2.9, 2.7 RPE -> VBRT 6-week post: 3.9, 4.7 RPE -> VBRT 12-week post: 5.6, 4.9	VBRT -> RPE 6-week post: 0.55, 0.51 VBRT-> RPE 12-week post: 0.21, 0.15 RPE -> VBRT 6-week post: 0.22, 0.32 RPE -> VBRT 12-week post: 0.35, 0.37		Yes
**Autoregulatory progressive resistance exercise**									
Mann et al. (2010)	College football players APRE: *n* = 12 LP: *n* = 11	6 × 3	Back squat Bench press	APRE pre: 196 ± 36.4, 134 ± 12.4 APRE post: 215.6 ± 20.4, 143.5 ± 10.5	LP pre: 215.4 ± 23.5, 154 ± 19.2 LP post: 219.2 ± 15.8, 153.9 ± 5.1	APRE: 10, 7.1 LP: 1.8, −0.1	APRE: 0.66, 0.83 LP: 0.19, 0	Repeated bench press repetitions 102 kg	No
**Flexible nonlinear periodisation**									
McNamara & Stearne (2010)	Untrained males and females. FNL: *n* = 8 NL: *n* = 8	12 × 2	Chest press Leg press	No value	No value	No value	No value	Standing long jump	Yes
Colquhoun et al. (2017)	RT male FNL: *n* = 14 NL: *n* = 11 age: 23.1 ± 6.3	10 × 3	Back squat Bench press Deadlift	FNL pre: 132.4 ± 34.2, 95.8 ± 20.1, 166.2 ± 40.6 FNL post: 148 ± 32.8, 102.3 ± 18.8, 181 ± 37.1	NL pre: 147.2 ± 30.7, 118 ± 20.8, 174.3 ± 25.4 NL post: 165.2 ± 25.4, 126.8 ± 21.2, 187.9 ± 29.2	FNL: 11.8, 6.8, 8.9 NL: 12.2, 7.5, 7.8	FNL: 0.47, 0.33, 0.38 NL: 0.64, 0.42, 0.50	Fat free mass; fat mass; bodyfat; Wilks coefficient; powerlifting total	Yes

**Note:**

*n*, sample size; RT, resistance-trained; RPE, rating of perceived exertion; PB, percent based; RIR, reps in reserve; APRE, autoregulatory progressive resistance exercise; LP, linear periodisation; FNL, flexible nonlinear; NL, nonlinear.

**Table 2:** Overview of intervention studies assessing an objective autoregulation training-method for manipulating training-intensity or training-volume upon 1-RM performance.

Reference	Training-group; Sample size (n); age (year)	Duration weeks × sessions per week	Resistance training exercise	Experimental pre: post: strength assessment (kg)	Comparison pre: post: strength assessment (kg)	Progression percent from pre: to post: both training groups (%)	Effect size from pre: to post: both training groups (Cohen`s d)	Other variables measured pre: and post:	Sets × reps equal between groups
**Velocity targets**									
Dorrell, Smith & Gee (2019)	RT male VBRT: *n* = 8 PB: *n* = 8 age: 22.8 ± 4.5	6 × 2	Back squat, Bench press, Strict overhead press, Deadlift	VBRT pre: 147.8 ± 25, 110.8 ± 15.2, 64.6 ± 8.5, 176.4 ± 31.4 VBRT post: 161.6 ± 27.1, 118.9 ± 14.6, 68.9 ± 7.9, 187.6 ± 30	PB pre: 131.9 ± 27.2, 94 ± 17.8, 58.1 ± 8.1, 176.9 ± 7.6 PB post: 143.8 ± 24.7, 98.4 ± 18.4, 61.7 ± 8.9, 182.1 ± 19.7	VBRT: 9.3, 7.3, 6.7, 6.4 PB: 9, 4.7, 6.2, 2.9	VBRT: 0.53, 0.54, 0.52, 0.36 PB: 0.46, 0.24, 0.42, 0.35	Countermovement jump (CMJ)	No
Orange et al. (2019)	Junior rugby players RT male VBRT: *n* = 12 PB: *n* = 15	7 × 2	Back squat	VBRT pre: 137 ± 18.5 VBRT post: 145 ± 16.6	PB pre: 136.6 ± 16.6 PB post: 145 ± 16.8	VBRT: 5.8 PB: 6.2	VBRT: 0.46 PB: 0.50	CMJ; 5-, 10-, 20-, 30-m sprint	Yes
Shattock & Tee (2020)	Rugby players RT male. VBRT: *n* = 10. RPE: *n* = 10.	Maximal strength: 6 × 4 Strength speed: 6 × 3	Back squat, Bench press	VBRT -> RPE pre: 137 ± 22.5, 102.2 ± 20 VBRT -> RPE 6-week post: 149+21.1, 112.3 ± 19.6 VBRT -> RPE 12-week post: 153.3 ± 20.7, 115.3 ± 19.5	RPE -> VBRT pre: 152.5 ± 26.6, 116.5 ± 17.3 RPE -> VBRT 6-week post: 158.5 ± 26, 122 ± 16.9 RPE->VBRT 12-week post: 167.3 ± 24.6, 128 ± 15.3	VBRT -> RPE 6-week post: 8.8, 9.9 VBRT-> RPE 12-week post: 2.9, 2.7 RPE -> VBRT 6-week post: 3.9, 4.7 RPE -> VBRT 12-week post: 5.6, 4.9	VBRT -> RPE 6-week post: 0.55, 0.51 VBRT-> RPE 12-week post: 0.21, 0.15 RPE -> VBRT 6-week post: 0.22, 0.32 RPE -> VBRT 12-week post: 0.35, 0.37	CMJ; 10-, 20-, 40-m sprint	Yes
**Velocity loss**									
Pareja-Blanco et al. (2017a)	RT male VL20: *n* = 12 VL40: *n* = 10 age: 22.7 ± 1.9	8 × 2	Back squat with smith machine	VL20 pre: 106.5 ± 12.2 VL20 post: 125.2 ± 12.3	VL40 pre: 104.5 ± 15.1 VL40 post: 118.6 ± 20.4	VL20 17.6 VL40 13.5	VL20 1.53 VL40 0.79	MRI; cross-sectional area vastus lateralis; fibre type analysis; CMJ; 20-m sprint	No
Pareja-Blanco et al. (2017b)	Soccer players RT male VL15: *n* = 8 VL30: *n* = 8 age: 23.8 ± 3.5	6 × 3	Back squat with smith machine	VL15 pre: 101.3 ± 18.8 VL15 post: 110.3 ± 14.3	VL30 pre: 100.2 ± 20.3 VL30 post: 106.5 ± 28.5	VL15 8.9 VL30 6.3	VL15 0.54 VL30 0.25	Average mean propulsive velocity; CMJ; 20-m sprint; Yo-yo intermittent recovery test	No
Galiano et al. (2020)	RT male VL5: *n* = 15 VL20: *n* = 15 age: 23.2 ± 3.2	7 × 2	Back squat with smith machine	VL5 pre: 97.7 ± 13.7 VL5 post: 108.2 ± 14.5	VL20 pre: 97 ± 13.9 VL20 post: 110.2 ± 18.4	VL5 10.8 VL20 13.6	VL5 0.74 VL20 0.81	CMJ; sprint 20 metre; AV, AV>1, AV<1	No
Sánchez-Moreno et al. (2020)	RT male. VL25: *n* = 15 VL50 N = 14		Prone grip pull up	VL25 pre: 108.4 ± 10.4 VL25 post: 114.3 ± 8.9	VL50 pre: 114.4 ± 20.8 VL50 post: 115.2 ± 19.8	VL25 5.4 VL50 0.7	VL25 0.61 VL50 0.04	APV _inc_ against absolute load common to pre: and post; MPV_best_ , MNR AV_MNR_	No
Rodríguez-Rosell et al. (2020)	RT male. VL10: *n* = 12 VL30: *n* = 13	2 × 8	Back squat with smith machine	VL10 pre: 100.8 ± 24.6 VL10 post: 116.6 ± 20.7	VL30 pre: 96.6 ± 14.7 VL30 post: 110.5 ± 15.2	VL10 15.7 VL30 14.4	VL10 0.7 VL30 0.93	20-m sprint; CMJ; muscle endurance; EMG; resting hormonal concentrations	No
Pareja-Blanco et al. (2020)	RT male. VL0: *n* = 14 VL10: *n* = 14 VL20: *n* = 13 VL40: *n* = 14	2 × 8	Squat	VL0 pre: 99.9 ± 19.1 VL0 post: 112.9 ± 19.9 VL10 pre: 96.7 ± 15.3 VL10 post: 113.6 ± 15.6	VL20 pre: 97.8 ± 23.2 VL20 post: 110.9 ± 19.6 VL40 pre: 94.5 ± 17.5 VL40 post: 105.6 ± 18.5	VL0: 13 VL10: 17.5 VL20: 13.4 VL40: 11.8	VL0: 0.66 VL10:1.1 VL20: 0.61 VL40: 0.62	Muscle hypertrophy and architecture of lateral vastus; tensiomyography; 20-m sprint; vertical jump; MVIC, fatigue test	No

**Note:**

*n*, sample size; VBRT, velocity-based resistance training; OT, optimum training; RT, resistance-trained; PB, percent based; VL5/15/20/30/40, velocity loss at 5%, 15%, 20%, 30% or 40%; AV, average velocity attained for all absolute training-intensities common to pre-test and post-test; AV > 1, average velocity for training-intensities that moved faster than 1 m/s; AV < 1, average velocity for training-intensities that mowed slower than m/s. AV_inc_, average mean propulsive velocity attained with all common external loads used during pre-training and post-training testing. MNR, maximal number of repetitions to failure in the pull-up exercise withoud external weight. MPVbest, fastest mean propulsive velocity attained withoud additional weigh in the pull-up progressive loading test. AV_MNR_, average mean propulisive veocity attainted agains the same number of repetitions to pre-test and post-test in the pull-up maximal number of repetitions test. MRI, magnetic resonance imaging. CMJ, countermovement jump. MVIC, maximal voluntary isometric contraction.

## Results

**Figure 2:**
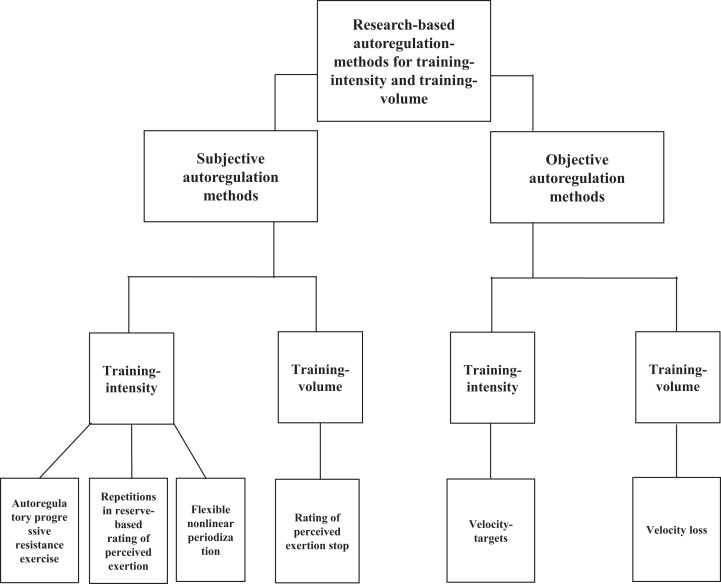
Different research-based autoregulation methods for resistance training. Subjective autoregulation methods are shown for manipulating the following training variables: training intensity, training volume, and training frequency. Objective autoregulation methods are shown for manipulating the following training variables: intensity, volume, and frequency.

Among the 14 studies attained, there were 30 experimental training groups comprised of 356 participants (Tables 1 and  2). For the subjective autoregulation methods, three studies investigated RIR and RIR based RPE, one study investigated autoregulatory progressive resistance exercise, and two studies investigated flexible nonlinear periodisation. Among the objective autoregulation methods, three studies investigated velocity targets and six studies investigated velocity loss (Fig. 2).

Nine different resistance exercises were used in the studies, which can be divided into three exercises for the lower extremity: different variations of squats (back squat, front squat and back squat in Smith machine), leg press and deadlifts, and four upper body exercises: bench press, chest press-in machine, overhead press and prone grip pull-ups. Six studies with seven experimental groups were trained with subjective autoregulation methods. One study, comprising of one experimental group trained with a subjective autoregulation method, who did not state pre-test and post-test values (McNamara & Stearne, 2010). 1-RM assessment from pre-test to post-test was performed on a total of six different resistance exercises.

For the RIR and RIR-based RPE load prescription experimental groups (Graham & Cleather, 2019; Helms et al., 2018a; Shattock & Tee, 2020), when training with the resistance exercise back squat, an ES between <0.8 and 0.5 was achieved for two experimental groups, while an ES < 0.5 was achieved for two other groups. Only one experimental group trained with the front squat, where they achieved an ES of 0.51 (Graham & Cleather, 2019). When training with the resistance exercise bench press, an ES between <0.8 and 0.5 was achieved for two experimental groups, while an ES < 0.5 was achieved for two other groups (Helms et al., 2018a; Shattock & Tee, 2020)

For the autoregulatory progressive resistance exercise experimental group (Mann et al., 2010), an ES between <0.8 and 0.5 was achieved for the back squat, while for bench press, an ES > 0.8 was achieved. For the flexible nonlinear periodisation experimental group (Colquhoun et al., 2017) an ES < 0.5 was achieved for all three exercises (back squat, deadlift and bench press).

Nine studies with eighteen experimental groups trained with objective autoregulation methods. 1-RM assessment from pre-test to post-test was performed on six different resistance exercises.

For the velocity targets experimental groups (Dorrell, Smith & Gee, 2019; Orange et al., 2019; Shattock & Tee, 2020), when training with the resistance exercise back squat, an ES between <0.8 and 0.5 was achieved for two experimental groups, while an ES < 0.5 was achieved for two other groups. Only one study assessed the ES on strict overhead press and deadlifts, where the ES was 0.52 and 0.36. When training with the resistance exercise bench press, an ES between <0.8 and 0.5 was achieved for two experimental groups, while an ES < 0.5 was achieved for one group.

For the velocity loss experimental groups (Galiano et al., 2020; Pareja-Blanco et al., 2020, 2017a, 2017b; Rodríguez-Rosell et al., 2020; Sánchez-Moreno et al., 2020), an ES > 0.8 was found in four groups, while eight reported ES of between 0.5 and 0.8. Only two groups (velocity loss of 50 and 30%) reported ES of respectively 0.04 and 0.25. When analysing it per resistance exercise all the squat studies reported ES over 0.5 except one that reported an ES of 0.25 (Pareja-Blanco et al., 2017b). When using the prone grip pull up ad resistance exercise an ES of 0.61 was found with the protocol of velocity loss of 25%, while with a velocity loss of 50% the ES was 0.04 (Sánchez-Moreno et al., 2020).

## Discussion

To the best of our knowledge, this is the first systematic review to summarise the literature regarding the effects of both subjective and objective autoregulation methods, which features intensity and/or volume adjustments for enhancing maximal strength during resistance-training interventions. Most of the subjective and objective autoregulation training groups achieved moderate to large increases in ES from pre-test to post-test. Autoregulatory progressive resistance exercise in the resistance exercise bench press was the subjective autoregulation method with the largest ES from pre-test to post-test (ES = 0.83) (Mann et al., 2010). Whereas RPE and flexible nonlinear periodisation in back squat had the largest percentwise progression from pre-test to post-test (11.8%) (Colquhoun et al., 2017; Helms et al., 2018a). Among the objective autoregulation methods for intensity, progressive VBT with velocity targets for the resistance exercise back squat achieved the largest ES from pre-test to post-test (ES = 0.55), while the bench press increased most in percent from pre-test to post-test in the same study (9.9%) (Shattock & Tee, 2020). In the objective autoregulation studies for intra-set volume, velocity loss 20% achieved the highest ES (ES = 1.53), and percent progression from pre-test to post-test 17.6% (Pareja-Blanco et al., 2017a).

### Rating of perceived exertion and reps in reserve

Helms et al. (2018a) and Graham & Cleather (2019) investigated the effects of RPE/RIR against percent-based and fixed-loading training groups and found that both autoregulation groups increased their 1-RM in all four exercises to a greater extent in ES and percentwise progression from pre-test to post-test than the traditional resistance-training approaches. However, in the study conducted by Helms et al. (2018a), strength differences between groups were small and not significant, but in the study by Graham & Cleather (2019), the RIR group increased the 1-RM to a greater magnitude than the fixed-loading group. A possible explanation for the absence of consistency in significant findings between the studies may be the length of the training intervention. Helms et al. (2018a) training intervention lasted for eight weeks, while in Graham & Cleather (2019) intervention, the participants trained for 12 weeks. During the first eight weeks of a resistance programme, most of the progression in strength is due to neural adaptations (Sale, 1988). In intermediate and advanced training, progress is limited to the extent of muscular adaptations the athlete can enhance. Since the participants in both studies were resistance-trained males, who were able to back squat and bench press 1.5 and 1.25 times body mass in the study of Helms et al. (2018a). While in the study of Graham & Cleather (2019) they were able to front squat and back squat 1.45 and 1.7 times body mass, the length of the training intervention in Helms et al. (2018a) may have been too short to successfully discriminate the possible benefits RPE could have on muscular adaptations and thus 1-RM. Furthermore, it is also speculated that the possible strength differences between the two studies may be attributed to the difference between the RIR-based RPE scale and the RIR-scale. Helms et al. (2018a) used the RIR-based RPE scale where RPE 9.5 states that no further repetitions could be completed, but slightly more load could be lifted. While Graham & Cleather (2019) used the RIR scale, where one RIR, means that the athlete could have completed one more repetition, while zero RIR, means zero repetitions in reserve.

Furthermore, Helms et al. (2018a) study had a variety of strengths in the execution of the methodology. Exercise selection, rest periods, sets, and repetition targets were identical between groups. All workouts were conducted in a laboratory at the same time each day under the supervision of the researchers. When a reported RPE score after a completed set fell outside the target RPE range, an adjustment to the load was made for the next set. Participants also recorded a 1–10 perceived recovery score by hand. This may have strengthened the study because it investigated significant differences in exhaustion between groups. No differences between groups were found before weeks 7 and 8. The RIR-based RPE group had a larger decrease in perceived recovery score from week 6 to 7, and thereafter an increase from week 7 to 8, than the percent-based group. The authors believed this could indicate that in week 7 where the intensity was the highest, the RIR-based RPE group overreached more than the percent-based group, and thus the RIR-based RPE had a more effective taper period than the percent-based group. The participants in the study by Graham & Cleather (2019) did not train in a laboratory. This was a clear limitation and could cause several issues since the researchers could not control that the prescribed training variables, such as intensity and volume, were interpreted correctly. This means that the participants in the RIR group could have overestimated or underestimated the prescribed RIR, and therefore, trained at different intensities than prescribed by the researchers. However, as previously mentioned in the introduction, Zourdos et al. (2016b) revealed that there was a strong, but not perfect inverse relationship between average concentric velocity and the RIR based RPE scale, for both experienced and novice lifters. Therefore, inaccuracies in estimating perceived effort during a set may be a limitation when implementing RIR or the RIR-based RPE scale into a periodized programme. Furthermore, It is also unclear if the participants in Graham & Cleather (2019) tested 1-RM in a laboratory or if they tested it in different gyms, which also could have affected pre-test to post-test values.

Even though training sessions were conducted outside a laboratory, this may have some positive aspects. With significantly greater progression in 1-RM for the RIR group, although all participants trained in the natural environment, this could have increased the ecological validity of the study. By comparing the studies, it seems like Helms et al. (2018a) controlled potentially confounding variables by performing the training and test in a laboratory. This may have led to high internal validity. The study by Graham & Cleather (2019) had a higher external or ecological validity since the participants conducted training in their training environment. Conducting studies with high internal validity followed by studies with high external validity may be a beneficial way to investigate a phenomenon within a paradigm. By controlling confounding variables, the researchers first get an overview of how manipulation of the independent variable causes’ changes in the dependent. Thereafter, carrying out studies in more natural environments may cause findings to be generalisable to the real-world population (Thomas, Nelson & Silverman, 2015). However, both studies were conducted on resistance-trained males, so that RIR-based RPE and RIR was more effective than percent-based training should not necessarily be generalised to untrained persons. The rationale for this speculation is evidence to suggest that RIR estimations on 1-RM could be less accurate in untrained populations (Ormsbee et al., 2019).

An interesting finding from both studies was that the autoregulation groups trained at significantly higher intensities than the control groups, even if the intensity was assigned to theoretically be the same. This may be related to the fact that RPE and RIR are subjective measures of effort that take into account that 1-RM is dynamic due to daily fluctuations. A desirable fluctuation that occurs with resistance training is that the practitioners enhance strength. Thus, it may seem that both RPE and RIR are better in accounting for the practitioners’ strength enhancement during a resistance-training programme than percent-based resistance protocols. Probably this important factor led to both studies favouring autoregulation methods for enhancing maximal strength in resistance-trained males.

### Flexible nonlinear periodisation/flexible undulation periodisation

In the studies of McNamara & Stearne (2010) and Colquhoun et al. (2017), both experimental groups that trained with a flexible nonlinear/undulation periodisation protocol increased significantly in maximal strength from pre-test to post-test. However, only the participants who trained the leg press in McNamara & Stearne (2010) study increased significantly more than the control group in the estimated 1-RM.

The findings between studies differ, and there could be several explanations for this. Firstly, the participants in McNamara & Stearne (2010) study were untrained males and females, while the participants in Colquhoun et al. (2017) study were resistance-trained males. Trained participants are physiologically different from untrained participants (Bishop, Jones & Woods, 2008). It could be more important for untrained than trained participants to have a certain degree of self-determination concerning intensity and volume for the workouts, based on fatigue and readiness. Another explanation for the different findings may be that participants in McNamara & Stearne (2010) study had a specific number of workouts during a four-week cycle that they could choose from, while participants in Colquhoun et al. (2017) study had to choose among three workouts each week. Therefore, it seems that participants in Colquhoun et al. (2017) had less opportunity to autoregulate intensity and volume in the workout based on daily fluctuations.

A possible limitation from the study of McNamara & Stearne (2010), was that they used an NSCA-protocol for estimating pre- and post-test 1-RM (Baechle & Earle, 2000). This was done by testing the participants for maximal repetitions performed, and thereby estimating the 1-RM from estimation tables. This could be problematic for several reasons. First, this assumes that there may be a linear association between the loads lifted and the repetition performed by the athlete. However, several studies have reported that this relationship is curvilinear (LeSuer et al., 1997; Mayhew, Ware & Prinster, 1993). Also, nearly every paper or study the NSCA estimation tables for 1-RM (Baechle & Earle, 2000) was based upon, used the back squat, power clean, or bench press to estimate 1-RM (Brzycki, 1993; Chapman, Whitehead & Binkert, 1998; Epley, 1985; Lander, 1985; Mayhew et al., 1992; Morales & Sobonya, 1996), which were not measured pre- and post in the study conducted by McNamara & Stearne (2010). McNamara & Stearne (2010) also did not account for the number of repetitions targeted. Since this is unclear, and perhaps not standardised, this may have influenced the validity of the pre-test and post-test measures. Another possible explanation for why Colquhoun et al. (2017) did not find significant differences between groups may be that the control group trained with the optimal order for the workouts. This means a hypertrophy-specific, power-specific, and strength-specific day. This speculation is based on the study from Zourdos et al. (2016a), who conducted a study comparing two daily undulating periodisation models on 1-RM in the squat, bench press and deadlift. The traditional daily undulating periodisation group trained the workouts in the order of hypertrophy-specific, strength-specific, and power-specific training, while the modified daily undulation periodisation group trained hypertrophy-specific, power-specific and strength-specific. The authors found that the modified daily undulation periodisation group increased the bench press significantly more in 1-RM compared to the traditional daily undulation periodisation group. The modified daily undulation periodisation group in Zourdos et al. (2016a), had the same exercise order as the traditional undulating periodisation group in Colquhoun et al. (2017). For practitioners, coaches and athletes, there may be no physiological benefits from performing this autoregulation method if the athlete is already training with a modified daily undulation periodisation protocol. However, the study revealed that 100% of the participants in the flexible daily undulation periodisation group completed the study, although two participants were removed from analyses due to extra activity. In the daily undulation periodisation group, only 69% of the participants completed the study. Therefore, like the authors pointed out in the discussion, using a flexible model may be more appropriate for the attendance of workouts, which is a long-term adaptation that could be positive for enhancing strength.

### Autoregulatory progressive resistance training

Mann et al. (2010) compared progression in estimated 1-RM between an autoregulatory progressive resistance exercise group and a linear periodisation group. It seems like this study has some limitations. Firstly, the participants were not randomised into groups. Therefore, it is a possibility that differences between groups in strength enhancement are due to unknown characteristics. The linear periodisation group was significantly stronger than the autoregulatory progressive resistance exercise group in the bench press and repeated bench press strength at baseline. It could be that the linear periodisation group did not progress from baseline to post-test because it was initially stronger at baseline. This leads to another limitation, which was that the baseline measurements for strength were not measured in a pre-test at a laboratory. It is therefore uncertain how the baseline strength assessment was performed. The study also did not state the warm-up protocol or whether the baseline testing and post-test were performed in the same location with the same equipment. It is also uncertain where the workouts were conducted and whether the prescribed training methods were performed correctly. The authors also state that there was no attempt to match volume and intensity and the intensity of training between the groups because the volume and intensity for the autoregulation progressive resistance exercise group were decided each day by the performance of the participants. The study also used estimated 1-RM with five or fewer repetitions to estimate the 1-RM. The investigators stated that they used a protocol, which was a commonly used protocol in the National Football League scouts to determine strength. This may be problematic for several reasons discussed in the flexible nonlinear periodisation part. Since the study has some methodical weaknesses, it would be wise not to draw clear conclusions from the findings. Besides, since controlling intensity and volume could be challenging during an intervention using this training protocol, the variables that cause any potential strength differences between groups cannot be said for sure. Eventual strength differences between groups may be explained by two factors separately or together. In the autoregulatory progressive resistance exercise group, the last two sets were performed to failure, and therefore participants probably trained with higher volume than the linear periodisation group. The other factor that probably can explain why the autoregulation progressive resistance exercise group progressed significantly higher in estimated 1-RM during the intervention was the decrease or increase in kg to the next workout based on repetitions performed during the last set in the prior workout. This could enable autoregulation for intensity progression between workouts based on the performance of the last set in each workout.

### Velocity targets

Dorrell, Smith & Gee (2019) and Orange et al. (2019) investigated the effects of velocity targets compared to percent-based training, and both autoregulation groups increased their 1-RM in all five exercises. However, in Dorrell, Smith & Gee (2019) study, the participants increased by almost 50% more than the percent-based group from pre-test to post-test. In Orange et al. (2019) study, the velocity target group increased similarly to the percent-based group. Some essential differences existed in the training protocols that probably could explain this. Perhaps the most obvious explanation was that participants in Dorrell, Smith & Gee (2019) study had a progressive increase in intensity, from 70% of 1-RM in week 1 to 95% of 1-RM in week 6 for the control group, while the velocity group had velocity targets that corresponded to this. Repetitions in both groups decreased from week to week, while intensity increased. Participants in Orange et al. (2019) study trained once a week at an intensity that corresponded to movement velocity at 80% of 1-RM and once a week at a movement velocity that corresponded to 60% of 1-RM for the experimental group, while the control group trained with percentages of 80% and 60% of 1-RM during the week. If participants achieved a mean velocity of ±0.06 m/s outside of the velocity target, the intensity was adjusted by ±5%. Sets and repetitions were static throughout the intervention. Therefore, the resistance protocol may not have been heavy enough and lacked adequate progression to allow autoregulation based on daily fluctuations to affect performance from pre-test to post-test.

Even though Dorrell, Smith & Gee (2019) demonstrated that VBT was superior when compared to percent-based training, their study had some possible limitations. The first was the use of concurrent augmented feedback during the set for each repetition performed. The use of feedback has been shown to improve performance when performing strength exercises (Argus et al., 2011; Weakley et al., 2019a). However, feedback from an objective measurement is one benefit of VBT, so even though this cofounding variable may have affected the results in this study, objective feedback can probably be a generalisable benefit to the real-world population. Another limitation was that the study used velocity thresholds based on groups, not the individual-based velocity threshold. Velocity differs individually against percentages of 1-RM. This means that the participants in reality probably exercised at different intensities, even though the velocity was the same. Another notable factor was that the VBT group trained with less volume than the percent-based group and still had a significantly higher increase in bench-press strength.

Shattock & Tee (2020), who compared velocity targets with RIR based RPE for adjusting training prescription during a 12-week intervention, demonstrated that the objective method was superior. This is an interesting finding and align with previous findings by Dorrell, Smith & Gee (2019) who demonstrated 9.3% progression in back squat and 7.3% progression in bench press from pre-test to post-test after a 6-week training-intervention. Similar Shattock & Tee (2020) demonstrated an 8.8% progression in squat and 9.9% progression in bench press 1-RM after 6 weeks of maximal strength training using velocity targets to prescribe intensity. The similarity in findings could be explained that velocity was prescribed with the same methods. Both studies used velocity thresholds based on groups for tracking and prescribing intensity. Since both volume and intensity were matched between groups, Shattock & Tee (2020) demonstrate the potential benefit objective devices for rating and tracking intensity may provide. These devices are accelerometers that provide augmented concurrent feedback on movement velocity during the set. Therefore, since feedback has been shown to have positive effects for acute power output (Argus et al., 2011; Weakley et al., 2019a), this may be a plausible explanation for why the objective autoregulation-group increased maximal strength to a greater extent than the subjective autoregulation-group during the training intervention. The velocity targets studies (Dorrell, Smith & Gee, 2019; Orange et al., 2019; Shattock & Tee, 2020) used validated accelerometer measuring tools during the training interventions. Where Orange et al. (2019) and Dorrell, Smith & Gee (2019) used a linear transducer (GymAware PowerTool, Kinetic Performance, 121 Technologies, Canberra, Australia). While Shattock & Tee (2020) used wearable accelerometers (PUSH, PUSH, Inc., Toronto, Canada) and iPads (Apple, iPad 4, iOS 10.3.3).

These devices have previously been investigated and shown an acceptable and comparable accuracy (Balsalobre-Fernández et al., 2016; Orange et al., 2020). However, studies have also shown that the PUSH devices for measuring and monitoring velocity may provide inaccuracies (Jovanovic & Jukic, 2020; Pérez-Castilla et al., 2019). Therefore, it is speculated, that the combination of innaccuracy (overestimating 1-RM) when predicting the 1-RM from the load-velocity relationship with different regression equations (Banyard, Nosaka & Haff, 2017; Hughes et al., 2019; Jukic et al., 2020; Lake et al., 2017; Ruf, Chéry & Taylor, 2018), together with potentially innaccuracies in velocity measurement devices (Jovanovic & Jukic, 2020; Pérez-Castilla et al., 2019), could be a possible limitation for accurate predicting intensity of the real 1-RM when using velocity targets to prescribe load.

### Velocity loss

Six studies (Galiano et al., 2020; Pareja-Blanco et al., 2020, 2017a, 2017b; Rodríguez-Rosell et al., 2020; Sánchez-Moreno et al., 2020) investigated the effect of nine different velocity loss threshold (0%, 5%, 10%, 15%, 20%, 25%, 30%, 40% and 50%) on increasing 1-RM or estimated 1-RM during training interventions (Table 2). Especially interesting are the findings of Pareja-Blanco et al. (2020, 2017a), who also measured hypertrophy of the lateral vastus, with the use of magnetic resonance imaging and lateral vastus muscle biopsies for muscle cross-sectional area and fibre-type analyses in Pareja-Blanco et al. (2017a), and B-mode ultrasonography to assess muscle cross-sectional are and architecture of the lateral vastus in Pareja-Blanco et al. (2020). Pareja-Blanco et al. (2017a) found that muscle hypertrophy significantly occurred in both the 20% and 40% velocity loss groups, but the 40% velocity loss group elicited greater hypertrophy for vastus lateralis and vastus intermedius. This is logical since a 40% velocity loss meant that the participant had to perform more repetitions, and therefore the intraset volume was higher for the 40% group. Also, the 2× myosin heavy chain isoform was unchanged in the 20% group but decreased in the 40% group. This is also a logical outcome of the training, since the 20% group trained with higher velocities. The findings from this study could indicate greater hypertrophy for the 40% group, while the 20% group maintained 2× fibres, which is important for explosive actions. A higher amount of 2× myosin heavy chain fibres means greater power output, which is important in many sports (Baechle & Earle, 2000). This is consistent with earlier research, where training to failure has been shown to decrease power output (Gorostiaga et al., 2012). However, the physiological cross-sectional area is shown to be positively correlated with maximal strength (Maughan, Watson & Weir, 1983). Therefore, the study illustrates that velocity loss with the use of a linear velocity transducer can affect physiological and neural adaptations which may be crucial for long term adaptations in maximal strength. Furthermore, there was no significant difference in 1-RM from pre-test to post-test for the Smith machine back squat. The 40% group performed 56% of the sets to failure, while the 20% group performed none of the sets to failure. Studies have shown that training with high volume reduced total testosterone levels and elevated cortisol levels (Fry & Kraemer, 1997; Hakkinen et al., 1988). Moreover, Izquierdo et al. (2006) showed that training to failure decreased insulin-like growth factor and increased its binding proteins. A high concentration of these hormones is a marker of stress (Borst et al., 2001; Elloumi et al., 2005). Therefore, training to failure in 56% of sets during an eight-week intervention may have affected hormonal levels and caused fatigue. It could have been that participants in the 40% group may have been fatigued, thus affecting post-strength assessment. Furthermore, Pareja-Blanco et al. (2020) demonstrated that 20% and 40% velocity loss thresholds both maximised hypertrophic adaptations, but a 40% velocity loss threshold also reduced the rate of force development capabilities of the participants.

Pareja-Blanco et al. (2017b) also found that a 15% group induced a similar increase in 1-RM Smith machine back squat strength from pre-test to post-test as the 30% group, even if the 30% group performed significantly higher volume during the intervention. This is consistent with the findings by Pareja-Blanco et al. (2017a), who also did not find differences between 40% loss and 20% loss in strength enhancement between groups in their intervention. Another consistent finding between the studies was that the 15% and 20% groups performed better than the 30% and 40% loss groups in the countermovement jump, which could be explained by the fact that the 20% group did not decrease in 2× fibres during the intervention (Pareja-Blanco et al., 2017a).

Galiano et al. (2020) found that both the 20% and 5% groups increased 1-RM in the Smith machine back squat from pre-test to post-test. Also, they found no significant differences in 1-RM between the groups, even though the 5% group performed only 32.6% of the repetitions performed by the 40% group. The participants in the study squatted 97.4 ± 13.6 kg during the pre-test. This means that they squatted only 1.3 times their body weight. It is therefore uncertain whether such a low intraset volume would have been appropriate if the participants were better enhanced in strength.

Overall, all six studies were quite homogenous in using only male participants, who in five of the studies, the participants squatted with a range from 94.5 to 114.4 kg in the pre-test. Also, mainly of the research for velocity loss upon squat performance was conducted on a Smith machine. Therefore, it could be interesting to see a study conducted on women, and also more experienced squatters, such as powerlifters. Also, more research is needed on other exercises with less stability than the Smith machine squat, because less stability has been shown to impact both peak forces and velocity (Van den Tillaar & Larsen, 2020). Furthermore, four of the six studies used estimated 1-RM instead of real 1-RM, which may have impacted the precision on 1-RM pre-test and post-test values (Galiano et al., 2020; Pareja-Blanco et al., 2017b; Rodríguez-Rosell et al., 2020; Sánchez-Moreno et al., 2020).

Altogether across the six studies, moderate velocity loss thresholds between 10% and 30% seem favourable when the goal is to enhance maximal strength (Table 2). The studies also demonstrate that velocity loss could be an effective method to autoregulate volume and thereby achieve different neural and physiological stimuli for enhancing maximal strength.

### Limitations

A limitation of this review was that only a few studies have investigated the effects of subjective and objective autoregulation methods for enhancing maximal strength during training interventions; therefore, it is challenging to answer the research question with great certainty and make generalisations from the studies analysed to the real-world population. Further research should investigate which resistance-training protocol is most effective for enhancing maximal strength among an individualised percent-based protocol, an RIR-based RPE protocol, or a VBT protocol using individualised and exercise specific velocity within subjects to autoregulate intensity. In addition, further research should investigate the effects of both subjective and objective autoregulation methods has for enhancing strength among females. Furthermore, only one study assessed the effects of autoregulatory progressive resistance exercise on enhancing maximal strength with some possible methodological limitations. Therefore, more studies may be needed to determine the longitudinal effect this autoregulation method has for increasing maximal strength. Also, studies assessing the effects of velocity loss thresholds on enhancing maximal strength were mainly done with the Smith machine back squat. Therefore, to increase the ecological validity, new studies should include how velocity loss thresholds may work on other free-weight exercises. Additionally, the inclusion of research which examined changes in estimated 1-RM may be a substantial limitation that must be acknowledged when interpreting the findings of this review, because such estimates are likely not typically valid.

## Conclusions

Overall, both subjective and objective autoregulation methods are effective for enhancing maximal strength when following a resistance-training protocol. It is speculated that this may be because both subjective and objective autoregulation methods could account for daily fluctuations in fitness, fatigue, and readiness of the athlete. An RIR-based RPE protocol seems to have small beneficial effects over a percent-based resistance protocol in the back squat, front squat, and bench press for increasing 1-RM. Therefore, it may be appropriate for athletes to increase maximum strength to use such a training protocol if they don’t have access to objective devices for tracking and monitoring intensity. Further, this review suggests that group-based VBT for monitoring intensity may be superior compared with RIR based RPE for increasing maximal strength in back squat and bench press during a training intervention. This could perhaps be explained because VBT provides objective augmented concurrent feedback during the resistance training exercise. Deciding intensity on the next workout based on the repetitions performed on a given intensity in the previous set could be an effective way of enhancing strength. However, only one study has assessed the effects of autoregulatory progressive resistance exercise on enhancing maximal strength. Also, interpretations regarding autoregulatory progressive resistance exercise should be made carefully, due to the methodological limitations from the study that investigated this training protocol. Despite no consistent findings regarding flexible nonlinear periodisation, choosing intensity or volume based on the athletes’ daily fluctuations seems to be an effective training method and could successfully be interpreted into an undulating training programme. If an undulation protocol with the order of hypertrophy-specific, power-specific, and strength specific is followed, it is speculated that implementing flexible nonlinear periodisation as a training protocol may have small physiological benefits. Using a validated measuring tool for velocity to track and autoregulate intensity and volume seems to be effective. An assumption for VBT to be a reliable and objective training method is that the athlete is performing the repetitions as fast as possible. If so, it may be beneficial to first make an individualised velocity profile for the athlete, since velocity at different intensities is individual among athletes. Thereafter, using velocity targets or stops to autoregulate intensity based on daily fluctuations could be an effective training protocol when the goal is to enhance strength. Furthermore, velocity loss is an effective training method for stimulating different neural and physiological adaptations, that could lead to long-term adaptations in maximal strength. Athletes who have access to such devices for measuring barbell velocity are encouraged to use these devices for monitoring changes in velocity, and thereby the possibility for adjusting the volume and intensity.

## Supplemental Information

10.7717/peerj.10663/supp-1 Supplemental Information 1 Hits of articles.

10.7717/peerj.10663/supp-2 Supplemental Information 2 PRISMA checklist.

10.7717/peerj.10663/supp-3 Supplemental Information 3 Rationale and contribution of the review.

10.7717/peerj.10663/supp-4 Supplemental Information 4 Search history.

## Additional Information and Declarations

### Data Availability

The following information was supplied regarding data availability: Raw data are available in the Supplemental Files.
