# Supplementary material for: Effects of subjective and objective autoregulation methods for intensity and volume on enhancing maximal strength during resistance-training interventions: a systematic review
Source: PeerJ. 2021 Jan 12;9:e10663. doi: 10.7717/peerj.10663 (PMC7810043; doi:10.7717/peerj.10663)
Supplement: Supplemental Information 1 [file peerj-09-10663-s001.pdf]

Training-method	Date searched:	Database	Keywords	Number of hits	Included in review	Search mode
Autoregulatory progressive resistance exercise	09.01.2020	SPORTDiscus	"Autoregulatory progressive resistance exercise"	4	1	Advanced search, Boolean/phrase
Rating of perceived exertion. Rating of perceived exertion with stop. Reps in reserve.	11.01.2020	SPORTDiscus	"Rating of perceived exertion" OR "reps in reserve" OR "RPE-stop" AND "1 repetition maximum"	3151	2	Advanced search, Boolean/phrase
Flexible nonlinear periodization	14.01.2020	SPORTDiscus	"Flexible nonlinear periodization"	8	1	Advanced search, Boolean/phrase
Velocity based resistance training	14.01.2020	SPORTDiscus	"Velocity based resistance training" OR "velocity-based training" OR "velocity loss" AND "1 repetition maximum" AND "linear position transducer" OR "linear velocity transducer"	86	2	Advanced search, Boolean/phrase
Autoregulatory progressive resistance exercise	09.01.2020	Google Scholar	"Autoregulatory progressive resistance exercise" AND "autoregulation" AND "1 repetition maximum"	1530	0	Advanced search, find all words
Rating of perceived exertion. Rating of perceived exertion with stop. Reps in reserve.	13.01.2020	Google Scholar	"Rating of perceived exertion" OR "reps in reserve" OR "RPE-stop" AND "1 repetition maximum" AND "autoregulation"	473	0	Advanced search, find all words
Flexible nonlinear periodization	14.01.2020	Google Scholar	"Flexible nonlinear periodization" AND "autoregulation" AND "1 repetition maximum"	90	1	Advanced search, find all words
Velocity based resistance training	15.01.2020	Google Scholar	"Velocity based resistance training" AND "autoregulation" AND "linear position transducer" OR "linear velocity transducer" AND "autoregulation"	2320	3	Advanced search, find all words, removed: patents AND quotes
Autoregulatory progressive resistance exercise	10.01.2020	PubMed	"Autoregulatory progressive resistance exercise"	2	0	Advanced search, all fields
Rating of perceived exertion. Rating of perceived exertion with stop. Reps in reserve.	13.01.2020	PubMed	"Rating of perceived exertion" OR "reps in reserve" OR "RPE-stop" AND "1 repetition maximum"	110	0	Advanced search, all fields
Flexible nonlinear periodization	14.01.2020	PubMed	"Flexible nonlinear periodization"	11	0	Advanced search, all fields
Velocity based resistance training	17.01.2020	PubMed	"Velocity based training" OR "velocity-based resistance training" OR "velocity loss" AND "linear position transducer" OR "linear velocity transducer"	85	0	Advanced search, all fields
