# Supplementary material for: Effects of subjective and objective autoregulation methods for intensity and volume on enhancing maximal strength during resistance-training interventions: a systematic review
Source: PeerJ. 2021 Jan 12;9:e10663. doi: 10.7717/peerj.10663 (PMC7810043; doi:10.7717/peerj.10663)
Supplement: Supplemental Information 2 [file peerj-09-10663-s002.docx]

| **Section/topic** | **#** | **Checklist item** | **Reported on page #** |
| --- | --- | --- | --- |
| **TITLE** | | |  |
| Title | 1 | Identify the report as a systematic review, meta-analysis, or both. | p. 1 Title: Effects o subjective and objective autoregulation methods for enhancing maximal strength during resistance-training interventions: A systematics review |
| **ABSTRACT** | | |  |
| Structured summary | 2 | Provide a structured summary including, as applicable: background; objectives; data sources; study eligibility criteria, participants, and interventions; study appraisal and synthesis methods; results; limitations; conclusions and implications of key findings; systematic review registration number. | p. 2-3. A structured abstract which includes the sections “Background”, “Objective”, “Materials and methods”, “Results”, and “Conclusion” is provided. |
| **INTRODUCTION** | | |  |
| Rationale | 3 | Describe the rationale for the review in the context of what is already known. | - An own rationale is provided as an own uploaded file. - p. 3-10. - Also, the introductory section we have given an overview of the autoregulation methods in resistance training. |
| Objectives | 4 | Provide an explicit statement of questions being addressed with reference to participants, interventions, comparisons, outcomes, and study design (PICOS). | p. 10.  Since studies suggest that both subjective and objective autoregulation methods could be used to prescribe training variables based on the daily fluctuations of the athlete, this study aimed to conduct a systematic review of the literature regarding the effects of subjective and objective autoregulation methods for intensity and volume on enhancing maximal strength during resistance-training interventions. |
| **METHODS** | | |  |
| Protocol and registration | 5 | Indicate if a review protocol exists, if and where it can be accessed (e.g., Web address), and, if available, provide registration information including registration number. | A protocol of this systematic review was not registered before the data collection. |
| Eligibility criteria | 6 | Specify study characteristics (e.g., PICOS, length of follow-up) and report characteristics (e.g., years considered, language, publication status) used as criteria for eligibility, giving rationale. | p. 10-11.  Study characteristics regarding the categories “training comparison group”, “pre- and post-test measure or estimation of 1-RM”, “article had to have a detailed description of total training volume”, “objective methods had to utilize a validated measuring tool to monitor velocity”, “only English-published studies”, “healthy participants”, “more than 4-weeks training intervention”. |
| Information sources | 7 | Describe all information sources (e.g., databases with dates of coverage, contact with study authors to identify additional studies) in the search and date last searched. | Figure 1 and p. 10.  We searched the electronic databases for eligible studies: SPORTDiscus, PubMed and Google Scholar. |
| Search | 8 | Present full electronic search strategy for at least one database, including any limits used, such that it could be repeated. | Se supplementary file. Also see page 11 where the full search strategy for PubMed are presented. |
| Study selection | 9 | State the process for selecting studies (i.e., screening, eligibility, included in systematic review, and, if applicable, included in the meta-analysis). | p.11.  Records were screened by one reviewer. |
| Data collection process | 10 | Describe method of data extraction from reports (e.g., piloted forms, independently, in duplicate) and any processes for obtaining and confirming data from investigators. | p. 11.  One reviewer extracted relevant data. A mail was sent to the corresponding author of Mcnamara et al (2010), Helms et al (2017), Shattock & Tee (2020) for getting relevant variables and information about the studies. |
| Data items | 11 | List and define all variables for which data were sought (e.g., PICOS, funding sources) and any assumptions and simplifications made. | table 1, 2 and page 11.  Primary outcomes are effect sizes calculated with Cohen`s d from pre- to post-test. Also, mean and standard deviation in 1-RM progression from pre- to post-test, together with percentwise progression in 1-RM. |
| Risk of bias in individual studies | 12 | Describe methods used for assessing risk of bias of individual studies (including specification of whether this was done at the study or outcome level), and how this information is to be used in any data synthesis. | p. 15-28.  The risk of bias was not calculated since the review did not use statistical analyses. However, the limited research and limitations in the presented studies are discussed, and an important part of this the review. |
| Summary measures | 13 | State the principal summary measures (e.g., risk ratio, difference in means). | table 1, 2 and page 11-14.  Effect sizes with Cohen. Small effect size = 0.2. 0.5 = medium effect size. 0.8 = large effect size. |
| Synthesis of results | 14 | Describe the methods of handling data and combining results of studies, if done, including measures of consistency (e.g., I^2^) for each meta-analysis. | n.a. |

Page 1 of 2

| **Section/topic** | **#** | **Checklist item** | **Reported on page #** |
| --- | --- | --- | --- |
| Risk of bias across studies | 15 | Specify any assessment of risk of bias that may affect the cumulative evidence (e.g., publication bias, selective reporting within studies). | p. 15-28.  This is discussed, and an important part of this review. |
| Additional analyses | 16 | Describe methods of additional analyses (e.g., sensitivity or subgroup analyses, meta-regression), if done, indicating which were pre-specified. | n.a. |
| **RESULTS** | | |  |
| Study selection | 17 | Give numbers of studies screened, assessed for eligibility, and included in the review, with reasons for exclusions at each stage, ideally with a flow diagram. | Figure 1. The databases SPORTDiscus, PubMed, and Google Scholar identified 7877 potential eligible studies. Studies sceened, assessed for elgibility and included are presented in figure and within the manuscript at page 11. |
| Study characteristics | 18 | For each study, present characteristics for which data were extracted (e.g., study size, PICOS, follow-up period) and provide the citations. | P 14 in tables 1 and 2 at p. 14. |
| Risk of bias within studies | 19 | Present data on risk of bias of each study and, if available, any outcome level assessment (see item 12). | The risk of bias is not presented as a statistical analysis, but discussed briefly in the discussion chapter p. 15-28. |
| Results of individual studies | 20 | For all outcomes considered (benefits or harms), present, for each study: (a) simple summary data for each intervention group (b) effect estimates and confidence intervals, ideally with a forest plot. | We have provided the outcomes percent progression, effect sizes, and mean and standard deviation in 1-RM or estimated 1-RM in tables 1- and 2 at p. 14. This is also presented at p. 12-14. |
| Synthesis of results | 21 | Present results of each meta-analysis done, including confidence intervals and measures of consistency. | n.a. |
| Risk of bias across studies | 22 | Present results of any assessment of risk of bias across studies (see Item 15). | n.a. |
| Additional analysis | 23 | Give results of additional analyses, if done (e.g., sensitivity or subgroup analyses, meta-regression [see Item 16]). | n.a. |
| **DISCUSSION** | | |  |
| Summary of evidence | 24 | Summarize the main findings including the strength of evidence for each main outcome; consider their relevance to key groups (e.g., healthcare providers, users, and policy makers). | We have summarised the main findings in the conclusion in the discussion chapter at p. 27-28, and abstract at page 2-3. Primary finding/conclusion was that both subjective and objective autoregulation methods may be effective for enhancing maximal strength. |
| Limitations | 25 | Discuss limitations at study and outcome level (e.g., risk of bias), and at review-level (e.g., incomplete retrieval of identified research, reporting bias). | p. 26  Several limitations are discussed within the limitation section in the discussion chapter. The main limitation is that only a few studies have investigated the effects of subjective and objective autoregulation methods for enhancing maximal strength during training interventions. Therefore, it may be challenging to answer the research question with great certainty and make generalisations to the real-world population. |
| Conclusions | 26 | Provide a general interpretation of the results in the context of other evidence, and implications for future research. | p. 27-28 A general conclusion was provided in the conclusion chapter.  Both subjective and objective autoregulation-methods may be effective for enhancing maximal strength during resistance-training interventions. |
| **FUNDING** | | |  |
| Funding | 27 | Describe sources of funding for the systematic review and other support (e.g., supply of data); role of funders for the systematic review. | We have provided a funding statement.  The authors did not receive funding’s for this work. |

*From:*  Moher D, Liberati A, Tetzlaff J, Altman DG, The PRISMA Group (2009). Preferred Reporting Items for Systematic Reviews and Meta-Analyses: The PRISMA Statement. PLoS Med 6(7): e1000097. doi:10.1371/journal.pmed1000097

For more information, visit: **www.prisma-statement.org**.

Page 2 of 2
