# Supplementary material for: Effects of subjective and objective autoregulation methods for intensity and volume on enhancing maximal strength during resistance-training interventions: a systematic review
Source: PeerJ. 2021 Jan 12;9:e10663. doi: 10.7717/peerj.10663 (PMC7810043; doi:10.7717/peerj.10663)
Supplement: Supplemental Information 3 [file peerj-09-10663-s003.docx]

1. **Rationale for conducting this systematic review.**

During the last decade, the use of autoregulation as a training method for prescribing volume and intensity has received a lot of attention among coaches, practitioners and athletes. Simultaneous, researchers in the sports science paradigm have validated different autoregulation methods and investigated the effect of these methods on enhancing maximal strength. Nevertheless, to the authors` knowledge, there is no systematic review that investigates the effect of these autoregulation methods on enhancing maximal strength during training interventions. Therefore, the rationale for conducting a systematic review on objective and subjective autoregulation methods for intensity and volume to enhance maximal strength during resistance training interventions, is to provide an overview for scientists, practitioners, athletes and coaches of the effects these autoregulation methods could provide on enhancing maximal strength.

1. **The contribution this systematic review makes to the knowledge about the body of evidence regarding autoregulation.**

This review could be of great interest for coaches, athletes, scientists and practitioners when choosing autoregulation methods for prescribing training intensity and volume into a periodized training programme. This is because this is the first systematic review that provides an overview on the different autoregulation methods on volume and intensity, and the effect these autoregulation methods has on enhancing maximal strength.
