# Supplementary material for: Effects of subjective and objective autoregulation methods for intensity and volume on enhancing maximal strength during resistance-training interventions: a systematic review
Source: PeerJ. 2021 Jan 12;9:e10663. doi: 10.7717/peerj.10663 (PMC7810043; doi:10.7717/peerj.10663)
Supplement: Supplemental Information 4 [file peerj-09-10663-s004.pptx]

## Slide 1
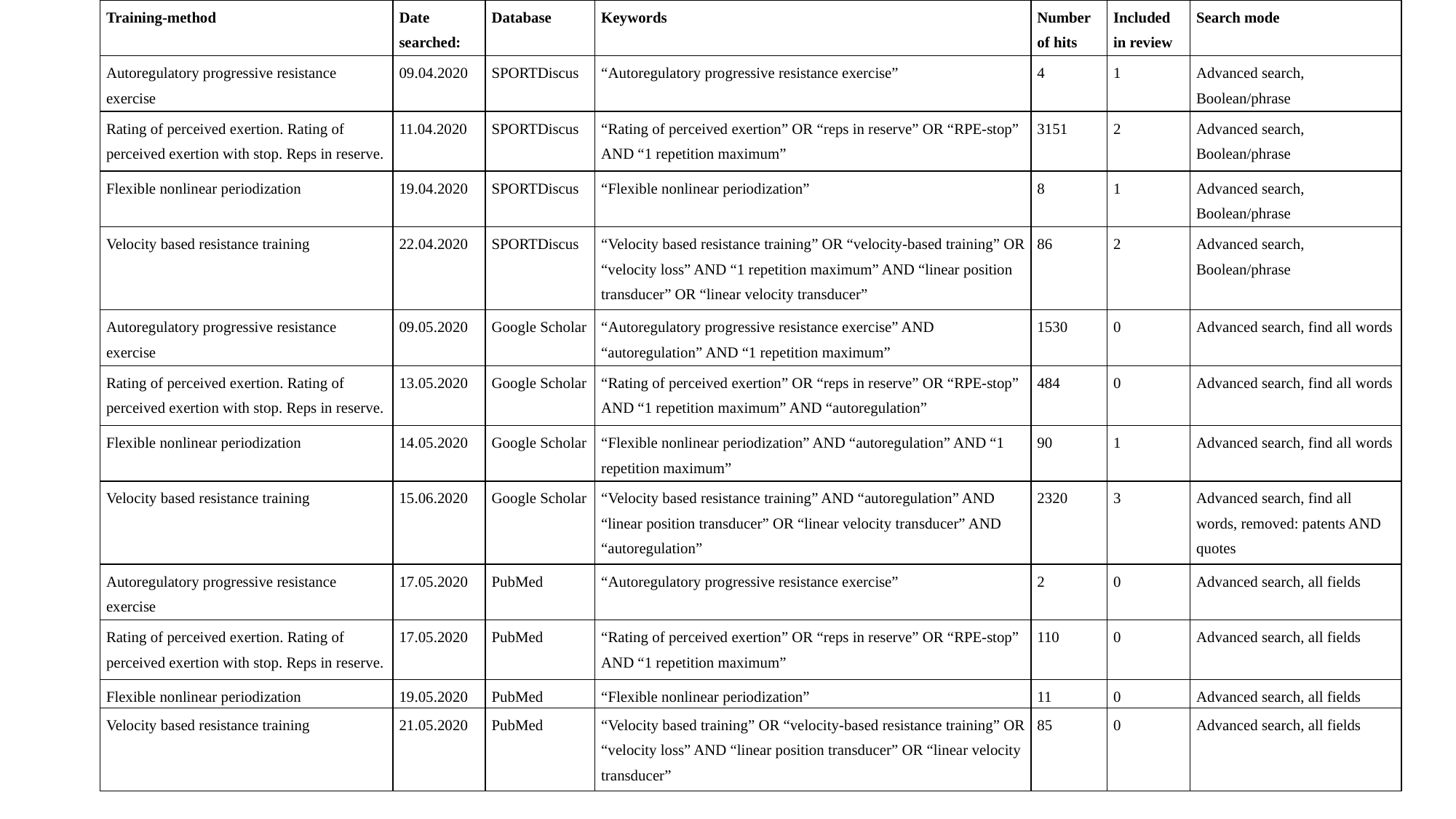

| Training-method | Date searched: | Database | Keywords | Number of hits | Included in review | Search mode |
| --- | --- | --- | --- | --- | --- | --- |
| Autoregulatory progressive resistance exercise | 09.04.2020 | SPORTDiscus | “Autoregulatory progressive resistance exercise” | 4 | 1 | Advanced search, Boolean/phrase |
| Rating of perceived exertion. Rating of perceived exertion with stop. Reps in reserve. | 11.04.2020 | SPORTDiscus | “Rating of perceived exertion” OR “reps in reserve” OR “RPE-stop” AND “1 repetition maximum” | 3151 | 2 | Advanced search, Boolean/phrase |
| Flexible nonlinear periodization | 19.04.2020 | SPORTDiscus | “Flexible nonlinear periodization” | 8 | 1 | Advanced search, Boolean/phrase |
| Velocity based resistance training | 22.04.2020 | SPORTDiscus | “Velocity based resistance training” OR “velocity-based training” OR “velocity loss” AND “1 repetition maximum” AND “linear position transducer” OR “linear velocity transducer” | 86 | 2 | Advanced search, Boolean/phrase |
| Autoregulatory progressive resistance exercise | 09.05.2020 | Google Scholar | “Autoregulatory progressive resistance exercise” AND “autoregulation” AND “1 repetition maximum” | 1530 | 0 | Advanced search, find all words |
| Rating of perceived exertion. Rating of perceived exertion with stop. Reps in reserve. | 13.05.2020 | Google Scholar | “Rating of perceived exertion” OR “reps in reserve” OR “RPE-stop” AND “1 repetition maximum” AND “autoregulation” | 484 | 0 | Advanced search, find all words |
| Flexible nonlinear periodization | 14.05.2020 | Google Scholar | “Flexible nonlinear periodization” AND “autoregulation” AND “1 repetition maximum” | 90 | 1 | Advanced search, find all words |
| Velocity based resistance training | 15.06.2020 | Google Scholar | “Velocity based resistance training” AND “autoregulation” AND “linear position transducer” OR “linear velocity transducer” AND “autoregulation” | 2320 | 3 | Advanced search, find all words, removed: patents AND quotes |
| Autoregulatory progressive resistance exercise | 17.05.2020 | PubMed | “Autoregulatory progressive resistance exercise” | 2 | 0 | Advanced search, all fields |
| Rating of perceived exertion. Rating of perceived exertion with stop. Reps in reserve. | 17.05.2020 | PubMed | “Rating of perceived exertion” OR “reps in reserve” OR “RPE-stop” AND “1 repetition maximum” | 110 | 0 | Advanced search, all fields |
| Flexible nonlinear periodization | 19.05.2020 | PubMed | “Flexible nonlinear periodization” | 11 | 0 | Advanced search, all fields |
| Velocity based resistance training | 21.05.2020 | PubMed | “Velocity based training” OR “velocity-based resistance training” OR “velocity loss” AND “linear position transducer” OR “linear velocity transducer” | 85 | 0 | Advanced search, all fields |
